# Risk Factors for Anemia among Brazilian Infants from the 2006 National Demographic Health Survey

**DOI:** 10.1155/2012/850681

**Published:** 2012-02-02

**Authors:** Tulio Konstantyner, Thais Cláudia Roma Oliveira, José Augusto de Aguiar Carrazedo Taddei

**Affiliations:** ^1^Discipline of Nutrology, Department of Pediatrics, Federal University of São Paulo, Rua Loefgreen 1647, 04040-032 São Paulo, SP, Brazil; ^2^Department of Epidemiology, Faculty of Public Health, University of São Paulo, Avenida Dr. Arnaldo 714, 01246-904 São Paulo, SP, Brazil

## Abstract

Iron deficiency is an important public health problem. An understanding of anemia risk factors is essential to informed health policies. We performed a cross-sectional study of 1,382 infants from the 2006 Brazilian National Survey on Demography and the Health of Women and Children. Mild and moderate anemia was characterised by hemoglobin levels below 11.0 and 9.5 g/dL, respectively. Rates for mild and moderate anemia were 25.9% and 9.9%, respectively. The logistic model included three risk factors for mild anemia—urban residence area (OR = 2.5; *P* = 0.004), fever in the past 2 weeks (OR = 2.4; *P* < 0.001), and age less than 12 months (OR = 1.7; *P* = 0.024). Strategies to control infant anemia should include health promotion and nutritional education for families from all socioeconomic levels. Lifestyle quality improvement based on adequate food consumption must be achieved by communities in all macroregions, and especially in urban areas.

## 1. Introduction

Iron deficiency is the most common and persevering nutritional disorder and continues to be an important public health problem worldwide [1]. Iron deficiency is responsible for the great majority of cases of anemia. Anemia has been used as a child health indicator due to its multifactorial detection characteristics and its demonstrated association with other health indicators in the paediatric age group, including premature weaning, malnutrition, parasitological and bacterial infections, and death [1, 2].

Specifically in children in the first years of life, hemoglobin (Hb) levels below 11 g/dL have been related to negative cognitive, social, and emotional effects that may lead to irreversible behavioural sequelae, even after appropriate treatment [3, 4]. In addition, severe anemia (Hb level below 5 g/dL) has been associated with an increased risk of child mortality [2, 5].

Researchers have shown high anemia prevalence on all continents and in both developing and developed countries [2]. In Brazil, studies have revealed anemia rates of approximately 50% in children younger than two years old, with intensity inversely proportional to age. However, the literature has provided results that identify individualised risks for infant anemia according to specific provinces, groups, and samples [6, 7].

The Ministry of Health of Brazil conducted the National Survey on Demography and the Health of Women and Children in 2006. That was the first Brazilian health survey to measure Hb levels based on a representative national sample to estimate the anemia prevalence in the country overall and to highlight sociobiological vulnerability areas [8].

Moreover, understanding anemia risk factors has helped health professionals to identify the groups that are more vulnerable to this morbidity earlier, indicating priorities in prevention and control action plans and in the allocation of available resources to improve child health promotion and care [9].

The objective of this study was to identify and quantify anemia risk factors in infants participating in a representative demographic health survey in Brazil and, consequently, to provide a comprehensive view to inform health strategies and policies and allow international comparisons.

## 2. Materials and Methods 

The present study used data from the National Survey on Demography and the Health of Women and Children (PNDS 2006), available on the Brazilian Ministry of Health website. That cross-sectional study aimed to determine the profile of fertile women and of children less than 5 years of age in the country's geopolitical macroregions. The study's methods, including sample design, data collection standards and procedures, data consistency, expansion technique for the complex sample, and ethical issues, have been reported elsewhere [8]. 

We analysed data from children less than 24 months of age from the PNDS 2006 sample. The initial population consisted of 1,902 infants. The following children were excluded: 108 had inadequate blood samples, 25 were not at home during the field activities, 37 whose families did not respond to our calls, and 350 whose guardians did not consent to the blood collection. Seventy-eight children were excluded from the multivariate analysis of mild anemia due to a lack of data on the included variables. 

Therefore, 1,382 children were selected and studied in the univariate analysis and 1,304 children were included in the multivariate analysis of mild anemia. To assess selection bias, we compared the sex, age, and rural-urban residence distributions of the children included in the multivariate models (*N* = 1, 304; 94.4%) with the group for which data for any of the included variables were missing (*N* = 78; 5.6%). No statistically significant differences were identified: *P* = 0.439, *P* = 0.517, and *P* = 0.733 for sex, age, and rural-urban residence, respectively. 

This sample size was sufficient to detect a minimum effect (odds ratio = 1.38) when anemia in the exposed group of children was compared with that of the control group of children, with a statistical power of 80% and a two-sided significance level (Type 1 error) of 0.05. 

Data were collected in the children's residences through interviews with the mothers or guardians, anthropometry, and blood samples drawn from the children using digital puncture. To measure Hb levels, the dried blood-spot technique was used for cyanmethemoglobin estimation. Whole blood was collected on filter paper and analysed using high-performance liquid chromatography (HPLC). Results of that technique using blood spot samples are comparable to standard laboratory techniques using venous blood samples for the measurement of Hb levels [10]. 

The outcomes were mild and moderate anemia, which were defined as Hb < 11.0 g/dL and Hb < 9.5 g/dL, respectively. The 11.0 g/dL cut-off value was based on international convention, whereas the other cut-off value is also used in the literature [11]. The anthropometric procedures adopted are recommended internationally, and *Z*-scores were used to quantify nutritional disorders. The benchmarks adopted for age and sex were those of the World Health Organization (WHO) 2006 [12]. 

To investigate associations between the variables, a chi-squared (*χ* ^2^) test was used [13]. The cut-off points for the dichotomous variables were based on recommended official or average values of the variables in the study sample [12, 14]. A serum retinol level of <0.7 *μ*mol/L was considered diagnostic of vitamin A deficiency. 

To adjust for confounding factors, a multivariate analysis was performed using a stepwise forward technique. The selection criteria for the explanatory and control variables for inclusion in the final logistic model were an association with mild anemia with *P* < 0.20. A maximum level of *P* = 0.05 was chosen to indicate a statistically significant association. In addition, to verify the adjustment of the logistic regression model, the Hosmer-Lemeshow goodness-of-fit test was used [15]. 

Hierarchical model for mild anemia risk factors was fitted to group the investigated variables according to each determination level, including socioeconomic factors, food consumption, child's health care conditions, child's nutritional status, presence of morbidity, and biological aspects [16]. 

Among the socioeconomic variables, “residence area (urban or rural)” was identified to compose the logistic model. Of the variables indicating individual child processes (nutritional status, morbidity, and biological aspects), “fever in the past two weeks” and “child age” were also selected to compose the model. 

Otherwise, vitamin A deficiency, the *Z*-score indicator of height-for-age (less than −2), receiving a basic-needs grocery package (a national food program for underprivileged citizens), cough in the past two weeks, and no maternal conjugal stability were among the selection criteria compounding the mild anemia logistic model (*P* < 0.20); however, these variables were not used because their statistical significance weakened when they were included in the model and they did not meet the permanence criteria. 

To adjust the effect of the selected variables, the following factors were also included in the model: macroregion, *per capita* family income, retinol serum level, and sex. These control variables were the last selected for inclusion in the final logistic model. 

All statistical procedures have been performed with the expansion technique for complex samples, which provided national representativeness for the five macroregions of Brazil (North, Northeast, Midwest, South, and Southeast) and the two residence areas (urban and rural). 

The Stata statistical software package Version 11.0 [17] was used to analyse the data and expand the sample. 

## 3. Results 

We estimated that 25.9% (CI 95%: 21.4; 30.3%) of the children in the country had mild anemia and 9.9% (CI 95%: 6.8; 12.9%) had moderate anemia in 2006. 

Table 1 shows the averages and prevalence estimates of the characteristics of all children at data collection. Thus, the average age was 11.6 months (CI 95%: 10.9, 12.4). The prevalence of living in an urban area was 80.0% (CI 95%: 76.0, 84.0). 

Table 2 presents the unadjusted odds ratios (ORs) of the investigated risk factors for mild and moderate anemia. Children living in an urban residence area were two times more likely to have mild anemia (*P* = 0.003) and three times more likely to have moderate anemia (*P* = 0.005). A value of *P* < 0.001 indicates that the odds of such a risk being the result of chance are less than 1/1,000. 

**Figure 1:**
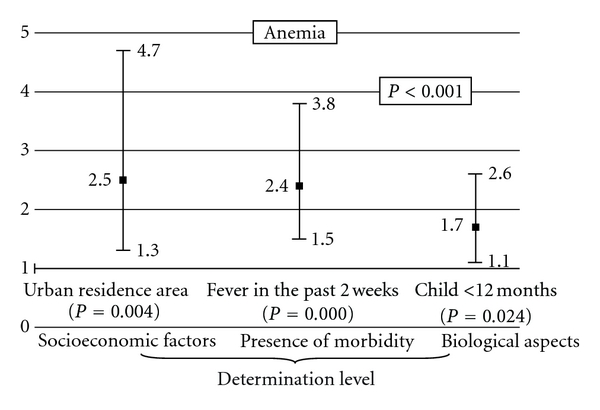
Multiple logistic regression of risk factors for mild anemia (Hb < 11.0 g/dL) of children younger than 24 months old who participated in the Brazilian National Survey on Demography and the Health of Women and Children (PNDS 2006), with odds ratios and their respective confidence intervals (95% CI). *Control variables: geopolitical macroregion, *per capita* family income, retinol serum level, and sex.

Figure 1 presents the multiple logistic model with OR and their respective CI of risk factors that exhibited statistically significant associations with mild anemia (*P* < 0.001) adjusted by control variables. 

This figure shows that urban residence area, fever in the past two weeks, and child age younger than 12 months are all independent risks for mild anemia. 

## 4. Discussion 

The results of this study indicate that in this countrywide representative sample, three factors were independently associated with mild anemia: living in an urban area, age less than 12 months, and fever in the past two weeks. 

Iron deficiency can produce cognitive functional limitations and social and emotional behavioural changes [3, 4]. Moreover, it has been determined to be either a cause or an effect of other health problems [2], which underscores the importance of health strategies to control and effectively prevent anemia. 

To do so, it is crucial to develop an understanding of the worldwide epidemiological profile of infant anemia. The rate of anemia has been shown to depend on child characteristics [6, 7, 9, 16, 18]. Studies have found many risk factors for anemia, including socioeconomic level, food consumption, health care, nutrition, morbidity, and biological factors. They are closely involved in a development process that results from several determining conditions [16]. 

The prevalences of mild and moderate anemia were approximately 25% and 10%, respectively. That result, although better than that of many other Brazilian studies [6, 9, 18], most likely reveals the constant difficulties health systems and policies have with protecting children from anemia. 

Of the factors in the final model, living in an urban area had a large impact on mild anemia, even after geopolitical macroregion and *per capita* family income were controlled. Traditionally, populations from rural areas usually have more difficultly accessing health services facilities and, consequently, with getting appropriate assistance, resulting in a higher frequency of health problems such as iron deficiency anemia [2]. 

In contrast with that perspective, our results show that infants living in urban areas had a higher risk of mild anemia. This is likely a reflection of the frequent migration from the fields to the cities in recent decades, resulting in people living under poor conditions in the slums of the country's metropolitan areas, and of the continuous lifestyle changes in urban areas: modernization, greater industrialisation of food, reduced awareness and knowledge of infants' food requirements, and the absence of a responsible adult care giver. Indeed, the quality of life and health of the urban population, especially in large cities, is subject to nutritional risks because of the accelerated pace of life and the greater availability of industrialised food [19, 20]. 

This finding also suggests that the Family Health Program, provided by the Ministry of Health since 1994, has improved health care access and primary care for families in rural areas and is effectively providing medicinal iron supplementation to control and prevent infant anemia [21]. 

The health promotion strategies used to control anemia are medicamentous iron supplementation, nutritional and health education, infectious disease control, and food fortification with bioavailable iron [2]. Although these strategies are being applied continuously in Brazil, the evidence identified in this study indicates that children living in urban areas are at higher risk of having health problems, based on anemia as an infant health indicator. 

Therefore, this finding indicates the need for integrated actions to improve infant health according to health promotion principles, which include addressing extreme poverty, hunger, disease, lack of water and sanitation, inadequate housing, and social exclusion and promoting gender equality, education, and environmental sustainability. In fact, those goals are also considered necessary to control iron deficiency anemia worldwide [2]. 

At the same time, fever in the past two weeks was identified as an associated factor for mild anemia; much like current fever has been associated with mild anemia in the literature [22]. Fever is a common symptom of acute and chronic inflammatory diseases, mostly infections, which have been associated with lower Hb levels. Existing anemia is aggravated by underlying inflammation, which leads to alterations in iron homeostasis, impaired erythrocyte proliferation, blunted erythropoietin response, and decreased erythrocyte half-life [23]. Moreover, several pro-inflammatory cytokines have been implicated in chronic inflammation anemia, including interleukin- (IL-) 1b, tumour necrosis factor-a (TNF-a), and IL-6 [22]. 

Age less than 12 months also was reported as a risk factor for mild anemia, confirming findings from several previous studies [7, 9, 16]. This may be explained by the high demand for iron to ensure accelerated physical growth during the first year of life, and by the difficulty mothers and guardians have ensuring adequate iron consumption after the sixth month of life, when stored iron is depleted and iron needs must be met through feeding [16]. 

Studies have shown an association between overweight and increased anemia prevalence in children and adolescents. Moreover, associations between anemia and overweight/obesity in children have been also reported in Brazil suggesting that excess food consumption and/or high metabolic gain potentially result in limited iron ingestion and storage [24, 25]. 

However, the anthropometric indicator of obesity used in this study did not show a statistically significant risk association with mild anemia. It is likely that this is a result of the several conditions of determination of obesity, of the sociocultural diversity, and of significant changes in food consumption in Brazil, which is known as the nutritional paradox [25]. 

Despite the results found in another study [9], our investigation did not identify a statistically significant association between weaning before 4 months of age and anemia or between ideal exclusive breastfeeding by age (children less than six months of age who were exclusively breastfed and children six months or older who were exclusively breastfed for the first 6 months of life) and anemia. This may be due to the mothers' difficulty with accurately remembering how long their children were exclusively breastfed (memory bias) and/or the time-modified confounding effect. These situations have been highlighted in the literature as barriers to collecting accurate data about exclusive breastfeeding [26]. 

In addition, low *per capita* family income, as an indicator of condition of extreme social deprivation, was not associated with anemia in this study, which was inconsistent with other studies [9, 16, 27]. Nevertheless, all risks identified for mild anemia were independent of *per capita* family income as an expression of purchasing power and availability of supplies per family unit. The risks were also independent of the Brazilian geopolitical macroregions, which are expressly distinguished according to economic development and social inequalities. 

The complex nature of anemia determinants motivated the multivariate statistical analyses in this study to identify situations in which children were independently more likely to have mild anemia based on a representative national sample. 

Therefore, the findings gain greater validity from the inclusion of other factors that potentially influence iron status in the multivariate analysis, providing the broad perspective necessary to understand events that are triggered by multiple risk factors [15]. As such, the effects were shown to be statistically significant when other variables, including geopolitical macroregion, *per capita* family income, serum retinol level, and sex, were controlled in the multifactorial model. 

However, it is worth noting that even though the study was performed under rigorous data collection and analysis criteria, sample loss may have affected the quality of the results, and hence, the findings of the present study may not accurately reflect reality. On the other hand, PNDS 2006 provided the only nationally representative data related to anemia in Brazilian infants; like other studies based on demographic health surveys, this study used the expansion technique of complex samples for all statistical procedures to ensure the legitimacy of the data [28]. 

It is also worth mentioning that PNDS 2006 was the first National Survey on Demography and Health performed in a continental country with a tropical climate to use the dried blood-spot technique to estimate Hb levels. Even based on controlled transport and storage conditions, environmental factors could cause deviations in the estimates of anemia prevalence. 

Moreover, because the analysis was based on an existing data set, we were limited to the use of variables found in the PNDS 2006. For instance, our study did not take into account the effect of early umbilical cord clamping after birth, which several studies have considered an important anemia determinant [29]. 

## 5. Conclusion 

This study indicates that strategies to improve infant health in Brazil should include health promotion and nutritional education for families from all socioeconomic levels, particularly in urban areas in all geopolitical macroregions. Lifestyle quality improvements based on adequate food consumption and appropriate care for children under 12 months of age must be achieved by communities and state authorities. 

Furthermore, the repeated characterisation of anemia as a serious public health problem by the scientific literature and the WHO [1, 2, 30], especially in developing countries such as Brazil, indicates that health managers and professionals should elaborate and execute more direct, more focused, and more integrated strategies to promote child health, involving all public health and education systems to effectively control iron deficiency in infants [31, 32]. 

In addition, the results reinforce that continuous changes in human mobility patterns and the urban lifestyle should aim to improve community participation and empowerment, which are core principles of the healthy cities movement in innovative health promotion [33]. 

 Finally, given the multiple factors involved in childhood anemia, including the role of food consumption and parental characteristics and lifestyle in urban areas, we recommend that qualitative studies of parental knowledge and perception of the need to prevent anemia in children be performed to determine behavioural characteristics that are susceptible to health and education interventions. 

## Figures and Tables

**Table 1:** Averages and prevalence of the characteristics of children younger than 24 months old who participated in the Brazilian National Survey on Demography and the Health of Women and Children (PNDS 2006), with their respective confidence intervals (CI 95%).

Characteristics (continuous variables)	*N*	*μ*	(CI 95%)	Characteristics (categorical variables)	*N*	P	(CI 95%)
Age (months)	1,382	11.6	(10.9; 12.4)	Urban residence area	1,382	80.0	(76.0; 84.0)
Maternal education (years)	1,357	6.7	(6.3; 7.2)	Receiving a basic-needs grocery package	1,381	7.8	(4.6; 10.2)
Paternal education (years)	1,308	8.3	(7.7; 8.9)	Eating any meat in the past week	1,363	58.3	(53.2; 63.3)
Home accommodations	1,381	5.0	(4.8; 5.2)	Eating liver in the past week	1,363	18.4	(15.0; 21.7)
Maternal age (years)	1,382	25.1	(24.5; 25.8)	Not having eaten anything in the past 24 hours	1,380	1.8	(0.4; 3.2)
*Per capita* family income (R$)	1,321	258.1	(222.7; 293.4)	No maternal conjugal stability	1,382	15.5	(11.5; 19.6)
Exclusive breastfeeding (days)	1,200	97.8	(89.1; 105.5)	Ideal exclusive breastfeeding according to age	1,200	30.5	(25.7; 35.3)
Hemoglobin level (g/dL)	1,382	11.7	(11.6; 11.8)	One or more siblings <5 years	1,382	34.9	(30.2; 39.4)
Retinol serum level (**μ**mol/L)	1,365	1.10	(1.05; 1.15)	Use of oral iron supplements	1,372	32.8	(28.1; 37.4)
*Z*-score indicator weight for age	1,358	0.12	(0.01; 0.22)	Fever in the past 2 weeks	1,380	25.5	(21.4; 29.6)
*Z*-score indicator height for age	1,336	−0.25	(−0.40; −0.10)	Cough in the past 2 weeks	1,381	33.6	(28.9; 38.3)
*Z*-score indicator weight for height	1,358	0.12	(0.01; 0.22)	Hospitalisation in the past 12 months	1,382	13.8	(9.8; 17.9)
Weight at birth (kilos)	1,348	3.28	(3.23; 3.33)	Male sex	1,382	55.2	(50.4; 60.0)

*μ*: average; P: prevalence; CI: confidence interval; R$: reals (Brazilian currency).

**Table 2:** Odds ratios of risk factors for mild anemia (Hb < 11.0 g/dL) and moderate anemia (Hb < 9.5 g/dL) of children younger than 24 months old who participated in the Brazilian National Survey on Demography and the Health of Women and Children (PNDS 2006), with their respective confidence intervals (CI 95%).

Risk factors		*N*	Mild anemia		Moderate anemia	
			Odds Ratios	*P* value*	Odds Ratios	*P* value*
*Socioeconomic factors*						
Residence area	Urban Rural	1,382	2.38 (1.35; 4.20) 1.00	0.003^†^	2.97 (1.39; 6.33) 1.00	0.005
Receiving a basic-needs grocery package	Yes No	1,381	1.91 (0.83; 4.41) 1.00	0.128	2.37 (1.06; 5.29) 1.00	0.035
Number of home accommodations	<5 ≥5	1,381	1.16 (0.70; 1.93) 1.00	0.599	1.10 (0.54; 2.26) 1.00	0.794
Per capita family income (R$)	≤258 >258	1,321	0.94 (0.58; 1.51) 1.00	0.789	1.36 (0.75; 2.46) 1.00	0.314
Paternal education (years)	<8 ≥8	1,308	1.40 (0.69; 2.81) 1.00	0.350	0.48 (0.12; 1.82) 1.00	0.278
Maternal education (years)	<4 ≥4	1,357	1.07 (0.67; 1.71) 1.00	0.768	1.05 (0.53; 2.08) 1.00	0.888
Maternal age (years)	<25 ≥25	1,382	1.09 (0.70; 1.71) 1.00	0.689	0.83 (0.49; 1.40) 1.00	0.481

*Food consumption*						
Exclusive breastfeeding (months)	≤4 >4	1,200	0.90 (0.56; 1.46) 1.00	0.682	0.64 (0.34; 1.18) 1.00	0.151
Not having eaten anything in the past 24 hours	Yes No	1,380	2.03 (0.45; 9.22) 1.00	0.360	1.78 (0.22; 14.53) 1.00	0.588
Ideal exclusive breastfeeding according to age	No Yes	1,200	0.74 (0.46; 1.19) 1.00	0.217	0.66 (0.35; 1.25) 1.00	0.198
Eating any meat in the past week	No Yes	1,363	1.37 (0.84; 2.25) 1.00	0.208	1.02 (0.57; 1.83) 1.00	0.942
Eating liver in the past week	No Yes	1,363	1.14 (0.70; 1.85) 1.00	0.607	0.55 (0.29; 1.04) 1.00	0.067

*Child's health care conditions*						
No maternal conjugal stability	Yes No	1,382	1.63 (0.81; 3.28) 1.00	0.175	1.08 (0.41; 2.86) 1.00	0.879
1 or more siblings <5 years	Yes No	1,382	0.92 (0.59; 1.43) 1.00	0.703	1.44 (0.81; 2.57) 1.00	0.215
Use of oral iron supplements	Yes No	1372	0.75 (0.48; 1.16) 1.00	0.200	0.90 (0.48; 1.68) 1.00	0.741

*Child's nutritional status*						
*Z*-score indicator weight-for-age	<−2 ≥2	1,358	1.36 (0.48; 3.84) 1.00	0.561	1.08 (0.27; 4.38) 1.00	0.910
*Z*-score indicator height-for-age	<−2 ≥2	1,336	0.46 (0.21; 1.00) 1.00	0.050	0.39 (0.14; 1.09) 1.00	0.072
*Z*-score indicator weight-for-height	>2 ≤2	1,325	1.11 (0.46; 2.64) 1.00	0.818	2.41 (0.80; 7.30) 1.00	0.119

*Presence of morbidity*						
Fever in the past 2 weeks	Yes No	1,380	1.95 (1.25; 3.04) 1.00	0.004^†^	1.53 (0.84; 2.79) 1.00	0.160
Cough in the past 2 weeks	Yes No	1,381	1.34 (0.89; 2.02) 1.00	0.159	1.29 (0.74; 2.26) 1.00	0.368
Vitamin A deficiency	Yes No	1,365	1.81 (1.04; 3.15) 1.00	0.035	1.77 (0.86; 3.65) 1.00	0.120
Hospitalisation in the past 12 months	Yes No	1,382	0.84 (0.48; 1.45) 1.00	0.530	0.65 (0.27; 1.45) 1.00	0.328

*Biological aspects*						
Weight at birth (kilos)	<2.5 ≥2.5	1,348	0.90 (0.37; 2.18) 1.00	0.815	0.21 (0.05; 0.94) 1.00	0.041
Child age (months)	<12 ≥12	1,382	1.62 (1.06; 2.48) 1.00	0.027^†^	1.11 (0.64; 1.93) 1.00	0.704
Sex	Male Female	1,382	1.11 (0.72; 1.73) 1.00	0.637	1.39 (0.73; 2.64) 1.00	0.311

CI: confidence intervals; *: chi-square (*χ* ^2^) test; R$: reals (Brazilian currency); ^†^: variables compounding the final logistic model.
